# Effects of *β*-Hydroxy-*β*-methylbutyrate Free Acid Ingestion and Resistance Exercise on the Acute Endocrine Response

**DOI:** 10.1155/2015/856708

**Published:** 2015-02-22

**Authors:** Jeremy R. Townsend, Jay R. Hoffman, Adam M. Gonzalez, Adam R. Jajtner, Carleigh H. Boone, Edward H. Robinson, Gerald T. Mangine, Adam J. Wells, Maren S. Fragala, David H. Fukuda, Jeffrey R. Stout

**Affiliations:** Institute of Exercise Science and Wellness, Sport and Exercise Science, University of Central Florida, Orlando, FL 32816, USA

## Abstract

*Objective*. To examine the endocrine response to a bout of heavy resistance exercise following acute *β*-hydroxy-*β*-methylbutyrate free acid (HMB-FA) ingestion. *Design*. Twenty resistance trained men were randomized and consumed either 1 g of HMB-FA (BetaTor) or placebo (PL) 30 min prior to performing an acute heavy resistance exercise protocol. Blood was obtained before (PRE), immediately after (IP), and 30 min after exercise (30P). Circulating concentrations of testosterone, growth hormone (GH), insulin-like growth factor (IGF-1), and insulin were assayed. Data were analyzed with a repeated measures ANOVA and area under the curve (AUC) was analyzed by the trapezoidal rule. *Results*. The resistance exercise protocol resulted in significant elevations from PRE in testosterone (*P* < 0.01), GH (*P* < 0.01), and insulin (*P* = 0.05) at IP, with GH (*P* < 0.01) and insulin (*P* < 0.01) remaining elevated at 30P. A significant interaction was noted between groups in the plasma GH response at IP, which was significantly higher following HMB-FA compared to PL (*P* < 0.01). AUC analysis revealed an elevated GH and IGF-1 response in the HMB-FA group compared to PL. *Conclusion*. HMB-FA prior to resistance exercise augments the GH response to high volume resistance exercise compared to PL. These findings provide further support for the potential anabolic benefits associated with HMB supplementation.

## 1. Introduction

*β*-Hydroxy-*β*-methylbutyrate (HMB), a leucine derived metabolite, has been demonstrated to augment strength and lean muscle gains when supplemented in conjunction with resistance training [1–4]. Recently, an 18% increase in strength gain was demonstrated following 12 weeks of HMB ingestion compared to placebo in experienced resistance trained men [1]. However, earlier studies of HMB were not consistent in demonstrating the efficacy of supplementation in regard to performance improvements [2, 5–7]. Many of the earlier studies used HMB formulated as a calcium salt (HMB-Ca); however, a new free acid form of HMB (HMB-FA) has been shown to yield higher plasma concentrations in a shorter amount of time compared to the calcium salt form [8]. The greater bioavailability of HMB-FA may provide greater benefits regarding its efficacy as a nutrient supplement used to enhance training adaptations.

While HMB has displayed robust anabolic/anticatabolic properties, the underlying mechanisms regarding its efficacy are not completely understood [9, 10]. HMB is known to increase muscle protein synthesis through mTOR signaling pathways while concurrently reducing proteolytic processes [10–13]. Furthermore, evidence also exists demonstrating HMB's ability to modulate specific cytokines and immune cells responsible for muscle tissue repair [14–16].

While several studies have observed an upregulation of certain target proteins in the mTOR pathway [17, 18], less is known about upstream components of the insulin-like growth factor-1 (IGF-1) dependent pathway [19]. Gerlinger-Romero and colleagues [19] observed increased growth hormone (GH) and hepatic IGF-1 expression, which corresponded to increased serum concentrations as a result of four weeks of HMB administration in male rats, suggesting that the effects of HMB may be mediated via IGF-1 activation of the mTOR cascade. While chronic HMB-Ca supplementation in elite teen athletes did not alter the GH/IGF-1 axis [5], to the best of our knowledge no studies are known that have investigated the effects of HMB-FA on the acute hormonal response in trained humans, which may result in proanabolic downstream effectors. Therefore, the purpose of the present study was to investigate the effect of acute HMB-FA ingestion on circulating concentrations of anabolic hormones following a heavy resistance exercise protocol in experienced, resistance trained men. We hypothesize that HMB-FA administration will augment the anabolic hormone response from a bout of resistance exercise resulting in increased activity of the GH/IGF-1 axis.

## 2. Methods 

### 2.1. Participants

Twenty resistance trained men (22.3 ± 2.4 y, 1.8 ± 0.1 m, and 7.3 ± 8.3 kg) volunteered to participate in this study. Participants were randomly separated into one of two groups: ingestion of HMB free acid (HMB-FA; *n* = 10) or ingestion of placebo (PL; *n* = 10). Following an explanation of all procedures, risks, and benefits, each participant gave his informed consent prior to participation in this study. The institutional review board for the protection of human subjects of the university approved the research protocol. For inclusion in this study, participants were required to have a minimum of one year of resistance training experience, particularly in the squat exercise. Participants were not permitted to use any additional nutritional supplements or medications while enrolled in this study. Screening for nutritional and hormonal supplements was accomplished via a health history questionnaire completed during participant recruitment.

### 2.2. Study Protocol

The investigation utilized a placebo-controlled, double-blind, randomized design. Participants reported to the laboratory on two occasions. On the first visit (T1), participants were tested for their one-repetition maximum (1-RM) on the barbell back squat, dead lift, and barbell split squat exercises. Participants were instructed to refrain from any form of exercise for a minimum of 72 hours prior to the resistance training bout (T2). On T2, participants completed a hypertrophy style lower-body resistance exercise session, which consisted of four sets of the barbell back squat, dead lift, and barbell split squat exercises. The barbell back squat exercise was performed with 80% of the participant's 1-RM and the dead lift and barbell split squat exercises were performed with 70% of the participant's 1-RM. Rest intervals were set at 90 s between each set and between exercises. Participants were encouraged to perform as many repetitions as possible up to 10 repetitions for each set. The duration of the acute exercise protocol was 45 minutes and total training volume, calculated as repetitions × load, was recorded for further analysis.

### 2.3. HMB Free Acid Supplementation

The HMB-FA supplement consisted of one gram of *β*-hydroxy-*β*-methylbutyrate in the free acid form (BetaTor, Metabolic Technologies Inc., Ames, IA), reverse osmosis water, debittering agent, flavor, stevia extract, and potassium carbonate. Each serving of placebo contained one gram of polydextrose and was identical to the HMB-FA supplement in appearance and taste. The HMB-FA and PL treatments were produced and supplied by Metabolic Technologies Inc. (Ames, IA). One serving of HMB-FA or PL was consumed 30 minutes prior to the exercise session. All HMB-FA and PL ingestion took place in the Human Performance Lab and was witnessed by one of the investigators.

### 2.4. Anthropometric Measurements

Prior to maximal strength testing, anthropometric measurements, including height, body mass, and body fat percentage, were conducted. Body mass (±0.1 kg) and height (±0.1 cm) were measured using a Health-o-meter Professional (Patient Weighing Scale, Model 500 KL, Pelstar, Alsip, IL, USA). All body composition measures were performed using standardized procedures previously described for collecting skinfold measurement from the triceps, suprailiac, abdomen, and thigh [20] and previously published formulas for calculating body fat percentage [21]. All skinfold measurements were performed by the same researcher using the same skinfold caliper (Caliper-Skinfold-Baseline, Model #MDSP121110, Medline, Mundelein, IL, USA).

### 2.5. Blood Measurements

During the T2 experimental session, blood samples were obtained before exercise (PRE), immediately after exercise (IP), and 30 min after exercise (30P). All blood samples were obtained using a 20-gauge Teflon cannula placed in a superficial forearm vein using a three-way stopcock with a male luer lock adapter. The cannula was maintained patent using an isotonic saline solution. PRE blood samples were drawn following a 15 min equilibration period prior to exercise. All IP blood samples were taken within one minute of exercise cessation. Following the resistance exercise protocol, subjects remained in the supine position for the full 30 min recovery phase prior to the 30P blood sample being drawn.

Blood samples were collected into two Vacutainer tubes, one uncoated serum tube and one K_2_EDTA plasma tube. A small aliquot of whole blood was removed from the K_2_EDTA plasma tube and used for determination of hematocrit and hemoglobin. The blood in the serum tube was allowed to clot at room temperature for 30 minutes and subsequently centrifuged at 3,000 ×g for 15 minutes along with the remaining whole blood from the K_2_EDTA plasma tube. The resulting plasma and serum were aliquoted into separate 1.8 mL microcentrifuge tubes and frozen at −80°C for later analysis.

### 2.6. Biochemical Analysis

Plasma HMB was analyzed by Metabolic Technologies Inc. by gas chromatography-mass spectrometry with previously outlined methods to confirm HMB appearance in the plasma [22].

Plasma testosterone (cat. number: KGE010), serum growth hormone (cat. number: DGH00), and plasma IGF-1 (cat. number: DG100) were assayed via commercial kits (R&D Systems Minneapolis, MN, USA). Serum insulin was also assayed via a commercial kit (RayBiotech, Inc., Norcross, GA, USA). Determination of serum immunoreactivity values was determined using a BioTek Eon spectrophotometer (BioTek, Winooski, VT, USA). To eliminate interassay variance, all samples for a particular assay were thawed once and analyzed in the same assay run by a single technician and intra-assay variance for the hormones was 5.8%, 5.3%, 4.1%, and 4.3% for testosterone, growth hormone, insulin, and IGF-1, respectively.

### 2.7. Statistical Analysis

Prior to analysis, all data were assessed to ensure normal distribution, homogeneity of variance, and sphericity. A 2 × 3 analysis of variance (ANOVA) (group [PL, HMB-FA] × time [PRE, IP, 30P]) was used to analyze all biochemical data. When appropriate, follow-up analyses included one-way repeated measures ANOVAs and LSD post hoc comparisons. In the event PRE values were significantly different, an analysis of covariance (ANCOVA) to analyze the effects of the intervention. The area under the curve (AUC) for all hormone concentrations was calculated by using a standard trapezoidal technique and was analyzed using paired Student's* t*-tests. An alpha level of *P* < 0.05 was used to determine statistical significance. All data are reported as mean ± SD. Data were analyzed using SPSS v22 software (SPSS Inc., Chicago, IL).

## 3. Results

**Figure 1:**
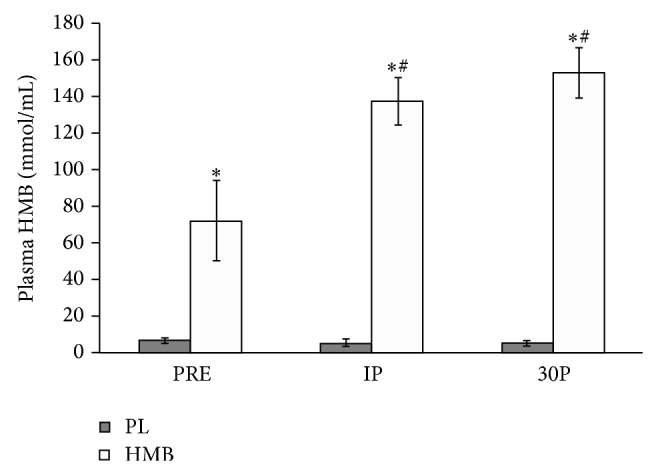
Plasma HMB concentration values for PL, HMB-FA, and time points at PRE, IP, and 30P. HMB was administered 15 min PRE blood draw.  ^*^Significantly elevated from PL and HMB-FA (*P* < 0.01); ^#^significantly elevated from PRE (*P* < 0.01).

The physical characteristics of the participants are presented in Table 1. No significant differences were noted in any of the anthropometric, strength, and experience level characteristics between groups. Plasma HMB concentrations were significantly elevated at IP (*P* < 0.01; 137.4 ± 38.7 mmol/mL versus 5.0 ± 5.8 mmol/mL) and 30P (*P* < 0.01; 153.0 ± 40.8 mmol/mL versus 5.0 ± 4.4 mmol/mL) for HMB-FA only (Figure 1). In addition, the total training volume per exercise session was not statistically different between groups. Plasma HMB concentrations have been reported earlier [23].

### 3.1. Hormone Responses

**Figure 2:**
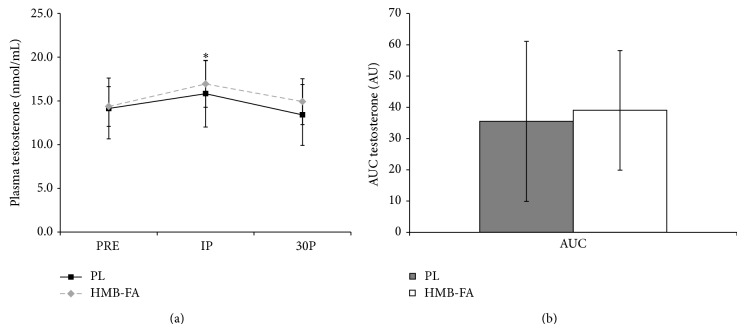
(a) Plasma testosterone concentration values for placebo (PL) and *β*-hydroxy-*β*-methylbutyrate free acid (HMB-FA) groups before exercise (PRE), immediately after exercise (IP), and 30 minutes after exercise (30P).  ^*^Both groups significantly elevated from PRE (*P* ≤ 0.01). (b) Area under the curve (AUC) analysis for plasma testosterone levels for PL and HMB-FA groups. Data were reported as mean ± SD.

Changes in testosterone concentrations can be seen in Figures 2(a) and 2(b). Significant increases in testosterone were seen in both groups at IP (*P* < 0.001) but returned to PRE concentrations by 30P (*P* = 0.533). No between group differences were noted. AUC analysis for plasma testosterone concentrations (Figure 2(b)) revealed no significant differences (*P* = 0.74) between the groups.

**Figure 3:**
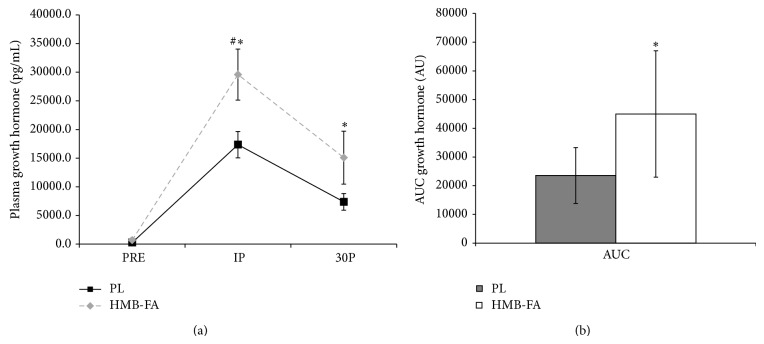
(a) Plasma growth hormone concentration values for placebo (PL) and *β*-hydroxy-*β*-methylbutyrate free acid (HMB-FA) groups before exercise (PRE), immediately after exercise (IP), and 30 minutes after exercise (30P).  ^*^Both groups significantly elevated from PRE (*P* ≤ 0.01). ^#^HMB-FA significantly elevated from PL (*P* = 0.05). (b) Area under the curve (AUC) analysis for plasma growth hormone levels for PL and HMB-FA groups. Data were reported as mean ± SD.  ^*^Indicating HMB-FA group is significantly greater than PL.

Growth hormone concentrations were observed to increase from PRE at IP (*P* < 0.001) and 30P (*P* < 0.001) in both groups in response to the exercise protocol (Figure 3(a)). However, the elevation in HMB-FA was significantly higher than PL at IP (*P* = 0.021), but no differences between the groups were seen at 30P (*P* = 0.10). AUC analysis revealed a significantly higher GH response (*P* = 0.02) in the HMB-FA group compared to PL (see Figure 3(b)).

**Figure 4:**
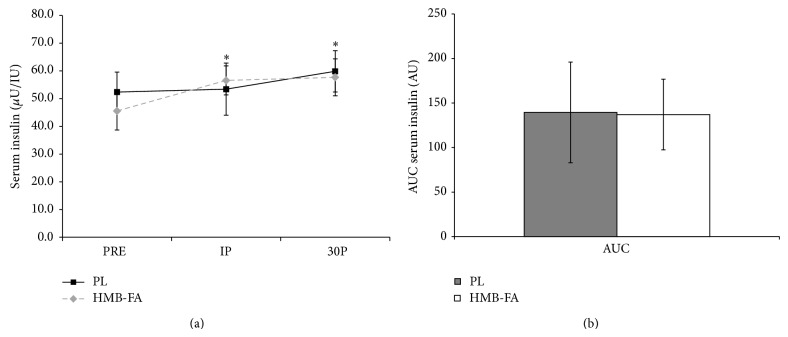
(a) Serum insulin concentration values for placebo (PL) and *β*-hydroxy-*β*-methylbutyrate free acid (HMB-FA) groups before exercise (PRE), immediately after exercise (IP), and 30 minutes after exercise (30P).  ^*^Both groups significantly elevated from PRE (*P* ≤ 0.01). (b) Area under the curve (AUC) analysis for serum insulin levels for PL and HMB-FA groups. Data were reported as mean ± SD.

Changes in insulin concentrations can be observed in Figures 4(a) and 4(b). Increases from PRE were observed in both groups at IP (*P* = 0.008) and 30P (*P* = 0.015), but no differences were observed between the groups.  Figure 4(b) depicts the AUC analysis on the insulin response during the exercise protocol. No difference (*P* ≤ 0.92) was observed between HMB-FA and PL.

**Figure 5:**
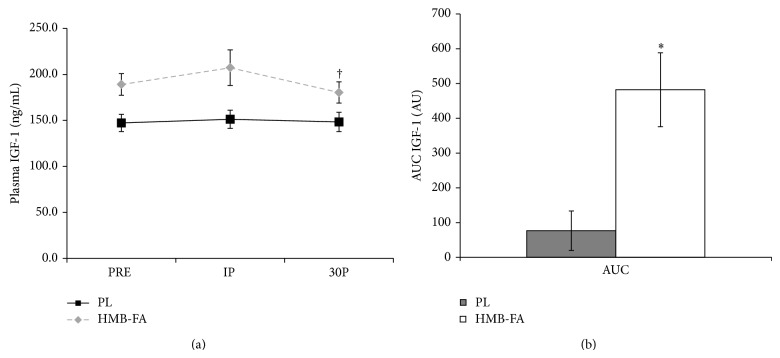
(a) Plasma insulin-like growth factor (IGF-1) concentration values for placebo (PL) and *β*-hydroxy-*β*-methylbutyrate free acid (HMB-FA) groups before exercise (PRE), immediately after exercise (IP), and 30 minutes after exercise (30P). ^†^Both groups significantly lower than PRE values. (b) Area under the curve (AUC) analysis for plasma IGF-1 levels for PL and HMB-FA groups. Data were reported as mean ± SD.  ^*^Indicating HMB-FA group significantly greater than PL.

Changes in IGF-1 concentrations from PRE to IP were not statistically different (*P* = 0.69) but significantly declined from IP to 30P (*P* = 0.015). IGF-1 values were significantly different (*P* = 0.012) between the groups at PRE. ANCOVA results showed no differences in IGF-1 concentrations (*P* = 0.31) in response to the workout. No differences were observed between the groups at IP or 30P (Figure 5(a)). However, AUC analysis (see Figure 5(b)) revealed a significant difference between HMB-FA and PL (*P* = 0.02) with HMB-FA ingestion resulting in a greater IGF-1 response following the resistance exercise protocol compared to PL. There was a correlation trend between plasma HMB AUC and IGF-1 AUC (*r* ^2^ = 0.585; *P* = 0.089).

## 4. Discussion 

The main findings of this investigation were that HMB-FA ingestion prior to resistance exercise can augment both the GH and IGF-1 response to a training session. To the best of our knowledge, this is the first study to demonstrate acute humoral alterations as a result of acute HMB-FA ingestion in trained men. The results of this study also provide evidence supporting a potentially greater anabolic response associated with HMB supplementation [1, 7, 17].

There is a well-documented dose-response relationship between training volume and the concomitant elevation in growth hormone secretion [24–27]. The high volume and short rest period employed during this study elicited a significant elevation in the GH and IGF-1 response in both treatment groups. Despite the similar training volumes between groups, HMB-FA ingestion immediately preceding the exercise protocol stimulated greater elevations in both GH and IGF-1 concentrations compared to PL. Previous studies have demonstrated that amino acids consumed alone or in conjunction with exercise can stimulate GH release to a greater magnitude when compared to placebo ingestion [28–31]. Thus, HMB-FA may alter the GH/IGF-1 axis through similar pathways as its leucine progenitor. While arginine, ornithine, and lysine [28–31] induce the largest elevations in GH among the amino acids, leucine has also been demonstrated to stimulate modest increases in GH concentrations [32]. However, amino acid ingestion does not appear to be as effective in enhancing the exercise-induced elevations in GH concentrations [31, 33, 34]. Our findings appear to be supported by a previous investigation examining a murine model [19]. In that study, increases in pituitary GH mRNA expression, hepatic IGF-1 expression, and serum IGF-1 concentrations were observed following four weeks of HMB supplementation [19]. Our results indicate that these effects appear to occur following an acute ingestion as well. Considering that one of the physiological roles of GH is to stimulate lipolysis [35], the significantly greater GH response in HMB-FA may provide further insight for the changes in body composition observed in several studies examining the efficacy of HMB [1, 2, 36].

The results of this study were unable to demonstrate any effect of HMB ingestion on the insulin response to the exercise protocol. This is in contrast with the study of Gerlinger-Romero and colleagues [19] who reported significant increases in resting insulin concentrations in HMB treated rats. These differences though are more likely a function of the prolonged supplementation protocol used by Gerlinger-Romero et al. [19], whereas we only investigated the hormonal responses from an acute ingestion and training session. While IGF-1 AUC values were significantly greater in the HMB-FA group in our study, the HMB-FA group was also elevated at baseline. Since the HMB-FA supplement was administered 15 min before the PRE blood draw, it is possible that HMB-FA is promoting IGF-1 secretion independently of GH stimulation.

Our findings in regard to the effect of HMB and resistance exercise on changes in the testosterone response suggest that acute ingestion does not augment the testosterone response to exercise. This is consistent with other studies that have examined the effects of prolonged supplementation of HMB and were unable to see any effect either with the free acid form [36] or as calcium salt [6]. Conversely, an amino acid based supplement containing HMB produced higher resting GH and testosterone levels following 12 weeks of resistance training with no changes in insulin or IGF-1 [2]. Additionally, this study implemented three acute exercise protocols throughout the 12 weeks to examine the acute response of supplementation. At week 6 and week 9 of supplementation, the testosterone response was elevated in the supplement group, whereas GH, IGF-1, or insulin responses were similar between the supplemental and control group. Thus, chronic supplementation with HMB may result in a more pronounced testosterone response, or it is possible that the other amino acids in the supplement (taurine, arginine) contributed to the observed hormonal response.

It is important to mention that the current investigation only administered one gram of HMB-FA (30 min prior to exercise) while other studies examining the effects of HMB on markers of recovery and muscle protein balance/synthesis have used three grams or chronic supplementation prior to the exercise session [1, 17]. However, it seems that one gram was sufficient in augmenting the transient exercise-induced elevation in GH compared to the placebo group. Currently, the literature is lacking in regard to the dose-response effects of HMB and future research is needed to explore optimal dosing strategies.

The rate of plasma HMB appearance we observed is in agreement with other studies [8, 13] suggesting that HMB-FA may be more beneficial than the calcium salt form when taken immediately before exercise. However, a recent study found no differences in absorption between HMB-FA and HMB-Ca in the animal model when both forms were administered in liquid suspension [37]. Thus, the gel form of HMB-FA may be responsible for its observed salient features. It is important to note that since we did not use HMB-Ca in the current study for comparison, further investigations examining various forms of HMB-FA and HMB-Ca are needed to translate findings to a trained human population.

In summary, these results appear to be the first examination suggesting that one gram of HMB-FA can promote a significantly greater postexercise increase in GH and IGF-1 compared to PL. This finding provides important contributions to our understanding of how HMB may affect endocrine function in humans. It appears that the HMB may modify the GH/IGF-1 axis while promoting no differences in circulating testosterone levels.

## Figures and Tables

**Table 1:** Characteristics of participants.

Characteristics	Placebo	HMB-FA
	*n* = 10	*n* = 10
Age (years)	23.8 ± 3.0	21.7 ± 2.0
Height (m)	1.78 ± 0.03	1.79 ± 0.08
Weight (kg)	85.7 ± 3.0	81.1 ± 12.7
Body composition (% fat)	13.0 ± 3.0	13.1 ± 4.8
Squat 1-RM (kg)	148.0 ± 3.0	135.9 ± 34.2
Training experience (years)	7.6 ± 3.0	5.95 ± 2.5

Values are means ± SD.
